# Tin Disulfide Nanosheet as Cathode Materials for Rechargeable Aluminum Ion Batteries: Synthesis, Electrochemical Performance, and Mechanism

**DOI:** 10.3390/molecules30081649

**Published:** 2025-04-08

**Authors:** Ruiyuan Zhuang, Xinming Tan, Yuxin Wang, Junhong Wang, Jianfeng Zhan, Jiangnan Yan, Jun Zhang, Lixiang Wang

**Affiliations:** 1 School of Mechanical and Electrical Engineering, Jiaxing Nanhu University, Jiaxing 314000, China; 2 School of Materials Science and Engineering, Jiangsu University, Zhenjiang 212013, China; 3 College of Biosystems Engineering and Food Science, Zhejiang University, Hangzhou 310027, China

**Keywords:** aluminum ion battery, tin disulfide, sheet structure, electrochemical performance

## Abstract

Aluminum ion batteries (AIBs) exhibit a promising development prospect due to their advantages such as high theoretical specific capacity, high safety, low cost, and sufficient raw material sources. In this work, nanosheet tin disulfide (SnS_2_) was successfully prepared using the hydrothermal method and then used as a cathode material for AIBs. The synthesized nano-flake SnS_2_ has a large size and thin thickness, with a size of about 900 nm and a thickness of about 150 nm. This electrode material effectively enhances the contact interface with the electrolyte and shortens the depth and travel distance of ion deintercalation. As an electrode, the battery obtained a residual discharge specific capacity of about 55 mAh g^−1^ and a coulombic efficiency of about 83% after 600 cycles. Furthermore, the first-principles calculation results show that the energy storage mechanism is the deintercalation behavior of Al^3+^. Based on model analysis and calculation results, it can be seen that compared with the position between two sulfur atoms, Al^3+^ is more inclined to be deintercalated directly above the sulfur atom. This study provides fundamental data for the large-scale preparation of AIBs using SnS_2_ as an electrode material and the application research of AIBs.

## 1. Introduction

With the continuous growth of global energy demand and the increasing depletion of fossil energy, efficient, environmentally friendly, and safe new energy batteries have emerged as an important direction for future development. Lithium-ion batteries (LIBs), as the current mainstream energy storage devices, are extensively utilized in various fields because of their high energy and power density. Nevertheless, due to issues such as the scarcity of lithium resources, potential safety hazards, and high costs, it remains challenging to apply LIBs on a large scale to meet the growing demand [1,2,3,4]. Therefore, there is a pressing need to develop new energy storage systems that offer advantages like low cost and high safety. In recent years, aluminum ion batteries (AIBs) have garnered significant attention from researchers. This is because they utilize inexpensive aluminum metal as the negative electrode, thereby reducing the cost of the battery. Additionally, they can achieve a high theoretical capacity (~2980 mAh g^−1^) through three-electron transfer during charging and discharging. Furthermore, the electrolyte used is a non-flammable ionic liquid, significantly enhancing safety [5,6,7]. Therefore, it is considered to be a new energy storage device with great application prospects. However, AIBs cathode materials still encounter issues like low capacity, low operating voltage, and poor cycle stability, which seriously hinder the development of AIBs. Therefore, there is an urgent need to find cathode materials with excellent electrochemical performance for rechargeable AIBs.

In recent years, transition metal chalcogenides have garnered significant interest in the research of AIBs, mainly because transition metal chalcogenides have the advantage of wide interlayer spacing, low electronegativity, and abundant resources. Their weak coulomb interaction with trivalent aluminum ions makes them more suitable for ion deintercalation, thus making them promising host materials for AIBs [8,9]. Among them, tin disulfide (SnS_2_), as a typical representative of transition metal sulfides, has a layered hexagonal CdI2-type crystal structure, which is composed of tin ions sandwiched between two layers of hexagonal close-packed sulfur atoms in an octahedral coordination form, presenting a sandwich configuration. The special crystal structure gives the electronic structure of SnS_2_ a higher carrier mobility (50 cm^2^ Vs^−1^) than other materials. Adjacent sulfur atoms are bound to each other by weak van der Waals forces, and the interlayer spacing is 0.5899 nm. This large interlayer spacing facilitates the insertion and extraction of ions. In addition, SnS_2_ has attracted the attention of researchers due to its high theoretical specific capacity (645 mAh g^−1^) and environmental friendliness [10,11,12,13]. Indeed, SnS_2_ has a wide range of applications in the field of energy storage. Chen prepared SnS_2_ nanosheets using a magnetron sputtering method and used them as electrode materials for LIBs, which exhibited 80% capacity retention after 200 cycles at 0.5 A g^−1^ and a high rate performance of 967 mAh g^−1^ at 1.0 A g^−1^ [14]. Leveraging the characteristic of large interlayer spacing in SnS_2_, which serves as a rapid transport pathway for potassium ions during charge–discharge cycles, Li employed it as the cathode in a potassium-ion battery, which also demonstrated outstanding electrochemical performance [15]. Based on the high capacity and two-dimensional structure of SnS_2_, Zhang used SnS_2_ as the anode materials for sodium-ion battery, and through rational electrode design, the battery achieved excellent rate performance and cycle stability [16].

It is well known that the physical and chemical properties of materials are closely related to their morphology. The same material with different morphological structures will exhibit many different physical and chemical properties that depend on the microstructure. In addition, constructing electrodes with different micromorphologies and improving the electron/ion transport kinetics of electrode materials by morphology regulation is also considered to be an effective means to improve the cycle life of batteries [17,18,19,20]. For example, nanosheet electrode materials have a high specific surface area, which can increase the contact interface with the electrolyte, shorten the depth and stroke of ion extraction and insertion, and reduce the degree of electrode polarization during high current charging and discharging [21,22]. Kien prepared nanowire-shaped and nanosheet-shaped nickel oxide with the hydrothermal method and conducted a comparative study on the electrochemical properties. The results showed that the performance of nickel oxide nanosheets was about ten times higher than that of nanowires. In addition, after three cycles in a strong alkaline environment, nickel oxide nanosheets showed an extremely high stability of almost 100% [23]. Gholamvand systematically studied the effects of nanosheet size and thickness on electrochemical parameters. The results showed that the electrochemical performance was inversely proportional to the length and thickness of the nanosheet [24].

Based on this, this study prepared SnS_2_ nanosheets through a simple one-step hydrothermal method and applied them as aluminum ion host materials in AIBs. As an electrode, the residual capacity of the AIBs after 600 cycles at 100 mA g^−1^ was about 55 mAh g^−1^, and the coulombic efficiency was close to 83%. In addition, this work combined the first-principles calculation to study the working mechanism of the composite electrode. The results showed that the energy storage mechanism was the deintercalation of Al^3+^ between each monolayer formed by S-Sn-S. Interestingly, compared with the embedding between two sulfur atoms, the formation energy of Al^3+^ embedded in the position directly above the sulfur atom is the smallest, at about −0.16 eV. The revelation of the mechanism helps to fundamentally improve the performance of tin disulfide-based electrodes. We believe that this work provides a useful reference for the research of high-performance flexible aluminum ion battery systems.

## 2. Results

**Figure 1:**
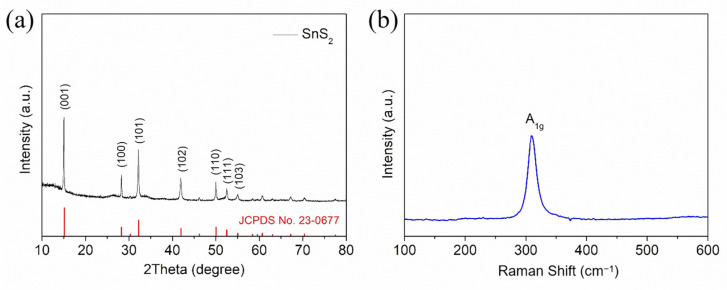
XRD pattern (**a**) and Raman spectrum (**b**) of SnS_2._

The crystal structure of the obtained sample was analyzed by XRD, and the results are shown in Figure 1a. It can be observed from Figure 1a that the diffraction peaks of the sample at 15.1, 28.2, 32.1, 41.9, 50.1, 52.5, and 55.1 correspond to the crystal planes (001), (100), (101), (102), (110), (111), and (103), respectively. All diffraction peaks can be attributed to hexagonal SnS_2_ (JCPDS No. 23-0677), which is consistent with previous reports [25,26]. No other impurity peaks were observed in the XRD spectrum, indicating that there are no other impurity phases in the SnS_2_ material. The Raman spectrum in Figure 1b provides more structural analysis for the synthesized sample. For the Raman spectrum of SnS_2_ nanosheets, the strong Raman peak at 311 cm^−1^ is usually considered to be the A_1g_ vibration mode of SnS_2_ [27]. XRD and Raman analysis show that SnS_2_ was successfully synthesized in one step through a solvothermal reaction.

**Figure 2:**
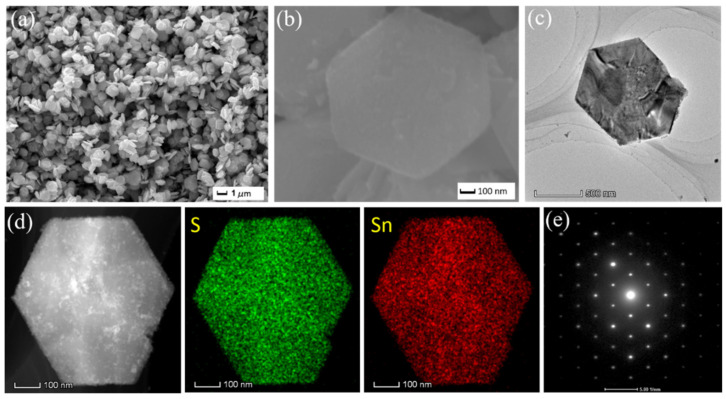
(**a**,**b**) SEM images of SnS_2_ with different magnifications; (**c**) TEM image of SnS_2_; (**d**) corresponding elemental mapping images of the SnS_2_ nanosheet; (**e**) SAED pattern of the SnS_2_ nanosheet.

Figure 2 shows the microscopic morphology and structure of the pure phase SnS_2_ sample. From the low-magnification SEM image in Figure 2a, it can be seen that the SnS_2_ sample has a good morphology and uniform size as a whole. It can be intuitively observed that SnS_2_ presents a large-scale two-dimensional nanosheet structure, with a size of about 900 nm and a thickness of about 150 nm. The high-magnification SEM image (Figure 2b) clearly shows the smooth and regular morphology of the SnS_2_ surface, further proving that the SnS_2_ sheet-like structure is distributed in a two-dimensional thin layer. The two-dimensional thin layer structure material has more obvious advantages than other structures in increasing the specific surface area and providing a high-speed carrier transmission channel. In order to further study the intrinsic morphology and structural characteristics of SnS_2_ nanosheets, the material was also characterized by TEM and SAED, and the results are shown in Figure 2c–e. The high-magnification TEM image in Figure 2c shows that the synthesized SnS_2_ has a typical sheet-like structure of quasi-hexagonal stacking. This is mainly because during the synthesis of SnS_2_, the growth of the crystal in the (001) crystal plane direction is restricted, but it grows rapidly in the six directions perpendicular to the (001) plane. Therefore, the synthesized SnS_2_ has a hexagonal nanosheet shape [28,29]. The EDS element mapping performed in Figure 2d is to analyze the element distribution of the material, and the results clearly confirm the clear composition distribution of Sn and S elements. In addition, the corresponding selected area electron diffraction (SAED) results are shown in Figure 2e. According to the SAED pattern, the sample has the results of different crystal orientations. Therefore, it can be reasonably concluded that the prepared SnS_2_ nanosheet is a single crystal with hexagonal symmetrical two-dimensional layered structure, where the incident electrons are parallel to the [0001] direction (c-axis). The sharp diffraction spots further indicate the high-quality crystalline structure of the as-synthesized SnS_2_ nanosheets.

**Figure 3:**
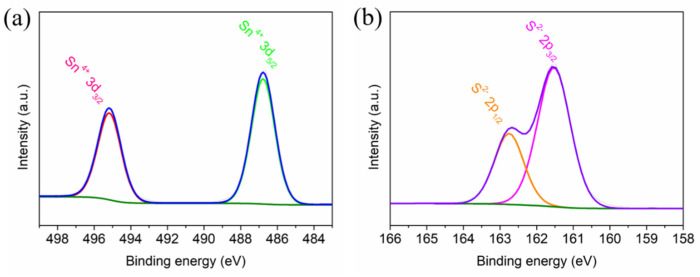
XPS high-resolution spectra of (**a**) Sn 3d; and (**b**) S 2p for the SnS_2_.

In order to explore the surface chemical state of the sample and its elemental valence state, the synthesized SnS_2_ was characterized by XPS analysis. As shown in Figure 3a, the high-resolution spectrum of Sn 3d is shown. The two super-strong peaks at 495.2 eV and 486.8 eV in the figure correspond to Sn 3d_3/2_ and Sn 3d_5/2_ of Sn^4+^ in SnS_2_ [30,31]. For the high-resolution spectrum of S 2p (Figure 3b), the peaks at 161.5 eV and 162.8 eV can be fitted to S 2p_3/2_ and S 2p_1/2_ of S^2−^ in SnS_2_, which indicates that the SnS_2_ phase is successfully formed [32,33].

**Figure 4:**
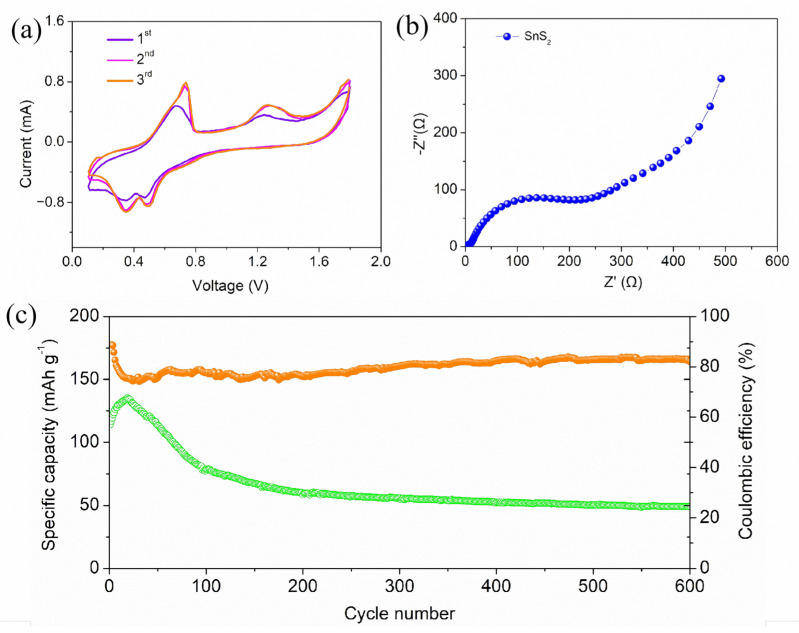
(**a**) CV curves of SnS_2_; (**b**) EIS spectra of SnS_2_; (**c**) cycling performance of SnS_2_ and SnS_2_ electrodes under 100 mAh g^−1^. Green and yellow curves represent capacity and coulombic efficiency, respectively.

The electrochemical performance of the SnS_2_ electrodes was studied by soft-pack batteries testing. Figure 4a shows the CV curves of the first three cycles of the SnS_2_ electrode. It can be seen that the CV curves of the electrode show two pairs of cathodic and anodic peaks. The positions of the cathodic peaks are approximately 1.28 and 0.63 V, respectively, while the positions of the anodic peaks are approximately 0.45 and 0.38 V. Interestingly, the first pair of peaks shown in the CV curve of the first cycle of the battery are not very obvious, but as the test continues, the peaks of the last two cycles appear, and the curves of the last two cycles have a high degree of overlap. In order to further study the electrode reaction kinetics, the electrode was also studied by electrochemical impedance spectroscopy (EIS). As shown in Figure 4b, the EIS test curve of the SnS_2_ electrode, the Nyquist curve contains a similar semicircle and a slant line. The diameter of the semicircle in the high-frequency region is related to the size of the charge transfer resistance (Rct) of the solid–liquid interface, while the Warburg impedance of ion diffusion in the electrode corresponds to the slant line in the low-frequency region [34,35]. After fitting analysis of the test data, it can be seen that the Rs value of the SnS_2_ electrode is 3.83 Ω, while the Rct value is 246.54 Ω. Figure 4c shows the constant current charge and discharge performance of the electrode. The battery was tested at a current density of 100 mA g^−1^. As can be seen, the residual discharge capacity of the SnS_2_ electrode after 600 cycles is about 55 mAh g^−1^ and the coulombic efficiency of the SnS_2_ electrode is about 83%. Interestingly, the curve consists of three parts, namely the initial rapid increase, the subsequent decrease, and finally maintaining a relatively stable state. Specifically, the AIBs showed an increase in capacity during the initial cycles. This phenomenon may be due to the activation step, which has also been observed in previous reports. After a few cycles, there is a noticeable decrease in capacity, which is a common issue in most AIB systems. However, the specific reason is still under investigation, and the most accepted explanation may be due to the formation of the solid electrolyte interface (SEI) layer or electrolyte decomposition [36,37]. In addition, to highlight the progress of this study, the electrode was compared with other published electrodes in similar fields, as shown in Table S1 (Supporting Information).

**Figure 5:**
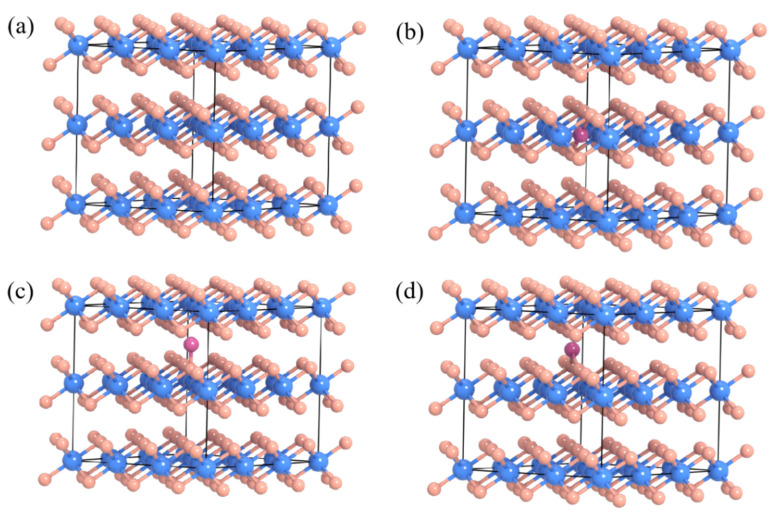
(**a**) Schematic diagram of the crystal structure of SnS_2_; (**b**) schematic diagram of the structure of aluminum atoms embedded in SnS_2_ crystals; (**c**) schematic diagram of the structure of aluminum atoms embedded directly above sulfur atoms between SnS_2_ layers; (**d**) schematic diagram of the structure of aluminum atoms embedded between two sulfur atoms between SnS_2_ layers.

In order to study the energy storage mechanism of the electrode, the crystal structure of SnS_2_ was finally analyzed by first-principles calculation. For this type of layered sulfide, there are usually two types of ion deintercalation channels. Therefore, this work calculated the deintercalation energy of the two channels and compared the results. Figure 5a is a schematic diagram of the crystal structure of SnS_2_, from which it can be seen that SnS_2_ has a sandwich layered structure. Each single layer is composed of three atoms, and two sulfur atoms sandwich the tin atom in the middle, and each single layer is stacked by tight covalent bonds. Figure 5b–d depict the three possibilities for aluminum ions to enter the SnS_2_ crystal. Figure 5b shows the aluminum ion entering the interior of the SnS_2_ crystal, that is, the single-layer space structure composed of tin atoms and sulfur atoms, and its formation energy is 0.78 eV. For the formation of energy to be positive, it means that the possibility of the reaction is low, so this embedding method can be ruled out. Secondly, we also considered the aluminum atoms entering from between the SnS_2_ layers, that is, the intermediate layers connected by van der Waals forces. We also considered two ways for aluminum atoms to enter the middle layer: one is for them to enter from directly above the sulfur atom (Figure 5c), and the other is for them to enter from between two sulfur atoms (Figure 5d). Calculations show that the formation energies for aluminum ions to enter from directly above and between two sulfur atoms are −0.16 and −0.34 eV, respectively. A smaller formation energy means a greater likelihood of the reaction occurring, so we conclude that during the charge and discharge process, aluminum atoms enter the SnS_2_ crystal through the gap between the layers, and the entry is directly above the sulfur atom.

**Figure 6:**
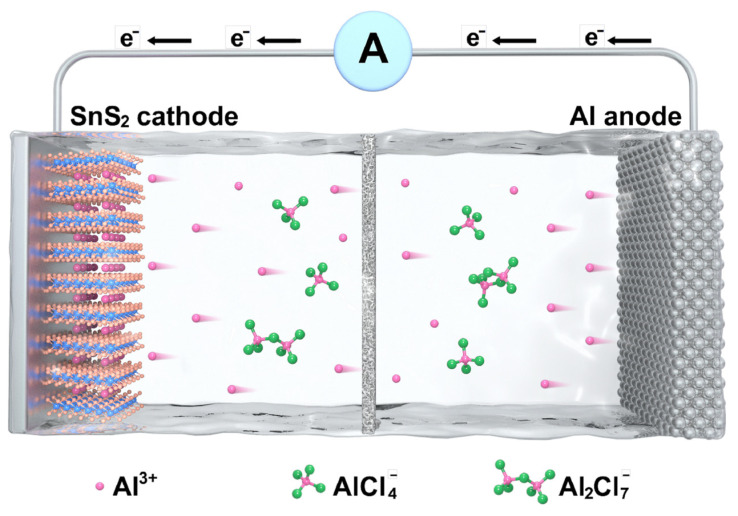
Schematic of the charging process in a Al/SnS_2_ battery.

Therefore, the schematic diagram of the charging and discharging process of SnS_2_ electrode is shown in Figure 6. Specifically, during the discharge process, Al^3+^ ions stripped from the anode are inserted into the intermediate layer of SnS_2_, while metallic Al combines with AlCl_4_^−^ in the electrolyte to form of Al_2_Cl_7_^−^. Reversible deposition and dissolution reactions can be observed on the anode side, mainly due to the composition of the electrolyte (AlCl_4_^−^ and Al_2_Cl_7_^−^). Hence, it can be concluded that aluminum ions undergo a deintercalation process during the charge discharge cycles, which can be summarized by the following equation:

$$Cathode:{Al}^{3+}+SnS_{2}+3xe^{-}\overset{discharge/charge}{↔}{Al}_{x}SnS_{2}$$

$$Anode:7\left[Al\right.{\left.{Cl}_{4}\right]}^{-}+Al\overset{discharge/charge}{↔}4\left[{Al}_{2}\right.{\left.{Cl}_{7}\right]}^{-}+3e^{-}$$

## 3. Materials and Methods

### 3.1. Synthesis of the SnS_2_

**Figure 7:**
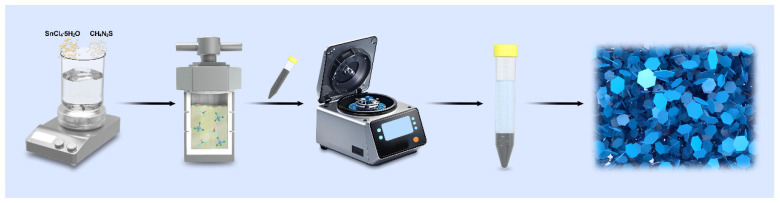
Schematic diagram of preparing SnS_2_.

First, weigh 2.5 mmol of SnCl_4_·5H_2_O (Shanghai Macklin Biochemical Co., Ltd., Shanghai, China) and 5 mmol of CH_3_CSNH_2_ (Sinopharm Chemical Reagent Co., Ltd., Shanghai, China), then pour the weighed two solid powder particles into a clean beaker. Next, add 80 mL of deionized water and stir continuously with a magnetic stirrer for 30 min to form a uniform and transparent solution. Secondly, transfer the prepared solution into the liner of a 100 mL high-pressure reactor, seal the sample and put it into an oven. Keep it at 170 °C for 12 h, and let it cool naturally to room temperature after the reactants react completely. Subsequently, wash the sample multiple times with anhydrous ethanol and deionized water through a high-speed centrifuge. Finally, vacuum dry the washed sample in a constant temperature drying oven at 60 °C for 12 h to obtain powdered SnS_2_. The detailed synthesis process of the sample is shown in Figure 7.

### 3.2. Synthesis of the Electrode

Initially, the prepared SnS_2_ powder, conductive carbon, and adhesive are mixed in a certain proportion, poured into a mortar and ground at room temperature with alcohol as solvent. The grinding process aims to make the active material and conductive carbon evenly mixed, and at the same time make the adhesive fully stretched, enabling it to serve as a bridge between the active material and conductive carbon, thereby facilitating better electron transfer. It should be noted that appropriate alcohol should be added during the grinding process to ensure that the electrode materials are fully ground and to achieve a slurry-like consistency. Once the mixed slurry reaches a desired consistency and humidity, the slurry is placed on a roller for sheeting. Ultimately, the slurry is rolled into an electrode sheet. Immediately following, the electrode sheet sample is placed in a culture dish and placed in a vacuum drying oven, and vacuum dried at 60 °C. After the sample is dried, the electrode sheet is taken out and cut into 12 mm discs with a punching machine, thus obtaining the SnS_2_ electrodes.

### 3.3. Battery Composition

The battery components tested in this work include electrodes, glass fiber separator papers, ionic liquid electrolytes, and aluminum foils. Among them, the ionic liquid electrolyte used in the experiment is prepared by [EMIm]Cl and AlCl_3_ (Shanghai Aladdin Biochemical Technology Co., Ltd., Shanghai, China) in a ratio of 1:1.3. The electrochemical performance test of the battery uses a soft pack battery, mainly because the ionic liquid electrolyte used in the experiment is highly corrosive to stainless steel metal. The electrode used in the test is the as-prepared SnS_2_ electrodes, and the current collector is a molybdenum sheet. The assembly process of the battery is to paste the electrode, glass fiber separator papers, and aluminum sheet together in turn, place them in an aluminum–plastic film, then drop in 250 uL of electrolyte and evacuate and seal through a thermoplastic machine. The final electrolyte addition and packaging steps of the batteries need to be carried out in a glove box filled with argon. The water oxygen value in the glove box must be less than 0.1 ppm. The sealed batteries can be tested after being placed in the test room for a certain period of time.

### 3.4. Characterization and Measurements

The molecular vibrational and rotational energy level structures of the synthesized materials were characterized by using the DXR instrument produced (Thermo Fisher Scientific, Waltham, MA, USA). During testing, a 523 nm laser with a power of 1 mW was employed. To acquire various physical and chemical information such as material morphology, composition, and crystal structure of the materials, the characterization instruments used in the experiment were the JSM-7800F scanning electron microscope and JEM-2100 transmission electron microscope. Additionally, to better analyze the element distribution and content of composite materials, the equipment was also equipped with an X-ray energy dispersive spectrometer.

The constant current charge–discharge tests of all batteries in the experiment were conducted on the NEWARE electrochemical testing system, with a test voltage window ranging from 0.1 to 1.8 V (relative to Al/AlCl_4_^−^). To investigate the electrochemical activity and reaction process of the electrodes, the batteries were also subjected to cyclic voltammetry (CV) tests using the PARSTAT MCEIS instrument of the Princeton Electrochemical Workstation (AMETEK, Newark, DE, USA). The test sweep rate was 0.5 mV s^−1^, and the scanning range was between 0.1 V and 1.8 V (relative to Al/AlCl_4_^−^). Additionally, internal resistance measurements of the batteries were conducted in this study, using the same instrument as for the CV tests, with sampling data ranging from 100 kHz to 10^−2^ Hz.

### 3.5. Computational Details

The spatial symmetry group of the SnS_2_ crystal structure in this study is P-3m1 (164). The supercell used in the computational simulation is a 3 × 3 × 2 SnS_2_ supercell, with a size of 10.94 × 10.94 × 11.80 Å3. The optimization simulation of the SnS_2_ supercell also employs the DFT-D3 approximate vdW method to calculate van der Waals interactions. We first conducted a complete structural optimization of the supercell model, and then we constructed aluminum ion embedding models and atomic replacement models at different positions for simulation calculations.

## 4. Conclusions

Herein, nanosheet SnS_2_ was successfully prepared by a simple one-step hydrothermal method and applied to AIBs. As an electrode material, its morphology and structure help to increase the contact interface with the electrolyte and shorten the ion migration distance. The assembled battery showed obvious electrochemical activity and long cycle life in the charge and discharge performance test. Specifically, the battery retained a discharge specific capacity of about 55 mAh g^−1^ after 600 cycles. The irreversible electrochemical properties and low Coulomb efficiency are mainly due to the low conductivity of the positive electrode material itself, the large energy density of Al^3+^ embedded and deintercalated in the electrode, and the relatively large electrochemical polarization of the electrode material. In addition, the energy storage mechanism model of Al^3+^ in the host material was simulated by density functional theory. The calculation results show that the energy storage mechanism of the battery is that Al^3+^ is deintercalated between the interlayers between single SnS_2_ layers, and Al^3+^ is deintercalated at the position directly above the sulfur atom. Although the electrochemical performance of the battery is not very satisfactory, the subsequent modification research can eliminate the electrostatic effect between Al^3+^ and the electrode material by extending the SnS_2_ interlayer spacing and introducing a higher conductive substrate material. This study provides basic data for the large-scale preparation of aluminum ion batteries using SnS_2_ as electrode material and the application research of aluminum ion batteries.

## Data Availability

Data are contained within the article.

## Supplementary Materials

The following supporting information can be downloaded at: https://www.mdpi.com/article/10.3390/molecules30081649/s1, Table S1 Performance comparison of SnS_2_ with several other transition metal dichalcogenides for application in AIBs. Refs [38,39,40,41,42,43,44,45,46,47] are cited in Supplementary Materials.
