# Supplementary material for: Tin Disulfide Nanosheet as Cathode Materials for Rechargeable Aluminum Ion Batteries: Synthesis, Electrochemical Performance, and Mechanism
Source: Molecules. 2025 Apr 8;30(8):1649. doi: 10.3390/molecules30081649 (PMC12029917; doi:10.3390/molecules30081649)
Supplement: Supplementary file 1 [file molecules-30-01649-s001.zip › molecules-3557055-supplementary.pdf]

Supplementary Information

Tin disulfide Nanosheet as Cathode Materials for Rechargeable Aluminum ion Batteries: Synthesis, Electrochemical Performance, and Mechanism

Ruiyuan Zhuang[1,2], Xinming Tan[1], Yuxin Wang[1], Junhong Wang[1], Jianfeng Zhan[1], Jiangnan Yan[1], Jun Zhang[1,3*], Lixiang Wang[1*]

1 School of Mechanical and Electrical Engineering, Jiaxing Nanhu University, Jiaxing 314000, PR China;

2 School of Materials Science and Engineering, Jiangsu University, Zhenjiang 212013, Jiangsu, China

3 College of biosystems engineering and food science, Zhejiang University, Hangzhou 310027, Zhejiang, China

* Correspondence: 11613006@zju.edu.cn; wlx@jxnhu.edu.cn

Table S1 Performance comparison of SnS_2 with several other transition metal dichalcogenides for application in AIBs

Cathode materials	Cycle number	Current density (mA g^{-1})	Specific capacity (mAh g^{-1})	Ref.
Graphene-VS_2	50	100	50	[1]
VS_4	120	400	129.24	[2]
Ni_3S_2@graphene	100	100	60	[3]
Mo_6S_8	50	12	70	[4]
Co_3S_4 microsphere	150	50	90	[5]
SnS porous film	100	200	70	[6]
Layered TiS_2	50	5	85	[7]
VS_4@rGO	100	300	80	[8]
WS_2@NCNFs	100	100	198	[9]
MoS_2	100	40	66.7	[10]
CoS_2/CNT	100	100	60	[11]
**SnS_2**	**600**	**100**	**55**	**This work**

1. Wu, L.; Sun, R.M.; Xiong, F.Y.; Pei, G.Y.; Han, K.; Peng, C.; Fan, Y.Q.; Yang, W.; An, Q.Y.; Mai, L.Q. A rechargeable aluminum-ion battery based on a VS_2 nanosheet cathode. *Phys. Chem. Chem. Phys.* **2018**, *20*, 22563-22568.

2. Xing, L.L.; Owusu K.A.; Liu, X.Y.; Meng, J.S.; Wang, K.; An, Q.Y.; Mai, L.Q. Insights into the storage mechanism of VS_4 nanowire clusters in aluminum-ion battery. *Nano Energy* **2021**, *79*, 105384.

3. Wang, S.; Yu, Z.; Tu, J.; Wang, J.; Tian, D.; Liu, Y.; Jiao, S. A novel aluminum-ion battery: Al/$AlCl_3$-[EMIm]Cl/Ni_3S_2@Graphene. *Adv. Energy Mater*. **2016**, *6*, 1600137.

4. Geng, L.; Lv, G.; Xing, X.; Guo, J. Reversible electrochemical intercalation of aluminum in Mo_6S_8. *Chem. Mater.* **2015**, *27*, 4926-4929.

5. Li, H.; Yang, H.; Sun, Z.; Shi, Y.; Cheng, H.-M.; Li, F. A highly reversible Co_3S_4 microsphere cathode material for aluminum-ion batteries. *Nano Energy* **2019**, *56*, 100-108.

6. Liang, K.; Ju, L.; Koul, S.; Kushima, A.; Yang, Y. Self-supported Tin sulfide porous films for flexible aluminum-ion batteries. *Adv. Energy Mater.* **2019**, *9*, 1802543.

7. Geng, L.; Scheifers, J.P.; Fu, C.; Zhang, J.; Fokwa, B.P.T.; Guo, J. Titanium sulfides as intercalation-type cathode materials for rechargeable aluminum batteries. *ACS Appl. Mater. Inter.* **2017**, *9*, 21251-21257.

8. Zhang, X.; Wang, S.; Tu, J.; Zhang, G.; Li, S.; Tian, D.; Jiao, S. Flower-like vanadium suflide/reduced graphene oxide composite: an energy storage material for aluminum-ion batteries. *Chemsuschem* **2018**, *11*, 709-715.

9. Yang, W.; Lu, H.; Cao, Y.; Jing, P. Single-/few-layered ultrasmall WS_2 nanoplates embedded in nitrogen-doped carbon nanofibers as a cathode for rechargeable aluminum batteries. *J. Power Sources* **2019**, *441*, 227173.

10. Li, Z.; Niu, B.; Liu, J.; Li, J.; Kang, F. Rechargeable aluminum-ion battery based on MoS_2 microsphere cathode. *ACS Appl. Mater. Inter.* **2018**, *10*, 9451-9459.

11. Zhang, K.Q.; Lee, T.H.; Cha, J.H.; Jang, H.W.; Shokouhimehr, M.; Choi, J.-W. Properties of CoS_2/CNT as a cathode material of rechargeable aluminum-ion batteries. *Electron. Mater. Lett.* **2019**, *15*, 727-732.
